# Progress in Bacteriology

**Published:** 1903-10-31

**Authors:** 


### Progress in Bacteriology.

Trypanosoma.—The section of tropical medicine of the British Medical Association[1] held a discussion on trypanosomiasis in the European and native. Manson referred to the probability of the parasite being found in India, and stated that for the examination of blood films it was best to spread moderately thin, stain with thionine, methylene blue, or Romanowsky's stain, and search with a $\frac{1}{6}$-inch objective. The blood is best examined when the fever is high. The clinical features of trypanosomiasis are undulant fever, erythema, œdema, muscular weakness, rapid irritable pulse, anæmia, breathlessness, enlargement of the spleen, mononuclear leucocytosis, and certain eye symptoms including external paralyses, choroiditis, iritis, cyclitis. Manson did not think that there was any causal relationship between trypanosoma and sleeping sickness, and the fact that the parasites had been found in the cerebro-spinal fluid of persons suffering from sleeping sickness was only indicative of the fact that a very large portion of the population was so infected. The inoculating agent, he thought, might prove to be a blood-sucking diptera, or a tick. Christy apparently believes in the relationship between trypanosomiasis and sleeping sickness, and indicates the tsetse fly as the causative agent. Castellani, too, believed that sleeping sickness was a trypanosoma infection although it was possible that different species of trypanosomes excited different pathological conditions in man. He summarised his arguments as follows:—The trypanosome is constantly present in the cerebro-spinal fluid of persons suffering from sleeping sickness. It is never found in the cerebro-spinal fluid of other diseases. The pathological changes of sleeping sickness and the mono-nuclear leucocytosis resemble these in trypanosomiasis. Sambon supported Castellani's views, and combated Manson's objection that the long incubation period was against the trypanosome theory. Other diseases due to protozoal infection, malaria, blackwater fever, sprue, hydrophobia, etc., had sometimes long periods of latency. Dutton and Todd described their researches on trypanosomiasis in West Africa. They had met with six cases in natives and one in a quadroon, and were inclined to think that the condition was not at all rare, occurring as a very mild disease which may in the native be unrecognised. Experimenting with various animals, they have only found trypanosomes in the horse. This is probably not the same species of parasite that is found in human beings, there being well marked differences in the disease produced by inoculation in lower animals. The animal most susceptible to inoculation with the human species of parasite is the goat. Experiments made with a view to transmitting trypanosomes from one animal to another by means of the bites of flies (stomoxys and glossina) were unsuccessful.

**Acute Rheumatism.**—Walker and Ryffel[2] give the following points as distinguishing the diplococcus rheumaticus from ordinary streptococci :—(1) Colour change produced in blood agar ; (2) growth in filtered culture of ordinary streptococcus ; (3) maximum growth in alkaline media ; (4) rapid production of acid ; (5) the production of rheumatic stigmata in inoculated rabbits ; (6) the production of a very active hæmolytic action upon red blood corpuscles. They have succeeded in isolating from the blood of infected animals an albumose which has the power of exciting hyperpyrexia. The rheumatic micrococcus produces in culture media relatively large quantities of formic acid and another fatty acid, and animals infected with these micro-organisms have these acids in the blood. Both the acids mentioned are present in the urine of patients suffering from acute rheumatism but not in normal urine. Under the salicylic acid treatment of acute rheumatism the formic acid in the urine is diminished. Streptococci do not form these acids. The action of the micro-organism is probably an oxidation of sarcolactic acid into formic and acetic acids.

**The Fusiform Bacillus.**—Fisher[3] reports two cases of ulcerative angina and stomatitis associated with the fusiform bacillus and spirillum of Vincent. Niclot and Marotte, in their description of the bacteriology of this condition, state that the fusiform bacillus is swollen at the centre with pointed extremities. It measures from $6\mu$ to $12\mu$ and may be either straight or curved. It is found in clumps and is motile, stains with the basic dyes, and is generally decolorised by Gram. There are involution forms, and the bacilli frequently occur in pairs. The spirilla are very long, $25\mu$ to $40\mu$, actively motile, and invariably decolorised by Gram's method. The superficial lesions are generally found to contain the bacillus together with the streptococcus, pneumococcus, colon, and Klebs Löffler bacillus ; in the deeper parts the bacillus is generally associated with the spirillum. Cultivations and inoculations have up to the present given negative results. The duration of the affection is from 10 days to six weeks, and without bacteriological examination it is difficult to distinguish from syphilis or diphtheria.

**Intravascular Antisepsis.**—Fortescue-Brickdale[4] has conducted a series of experiments in order to test the toxicity of various antiseptics when injected into the veins of rabbits, and secondly to discover if any of them produced an influence on an artificially produced septicæmia. The strength of the drug employed is expressed in relation to its proportion per 100 grammes body-weight. With perchloride of mercury the animals died before the blood could be raised to any appreciable degree of toxicity, and the same result was obtained with oxycyanide of mercury and protargol. Chinosol and formic aldehyde mixed is too toxic for practical purposes, but either may be injected intravenously so as to produce a solution which would have an inhibitory action in vitro. Tested on artificially infected animals ; it was found that animals injected daily with non-toxic doses of oxycyanide of mercury, formic aldehyde, chinosol, protargol or tauxocholate of sodium are not thereby protected from the usual effects of a previous inoculation of virulent anthrax, and that chinosol and formic aldehyde in large doses so depress rabbits infected with the pneumococcus that they die sooner than untreated animals.

[1] Brit. Med. Jour., Sept. 19, 1903. [2] Ibid. [3] Amer. Jour. Med. Sci., Sept. 1903. [4] Lancet, Jan. 10, 1903.

